# Engineering *Streptococcus thermophilus* for heterologous gene expression of cell envelope proteases from lactic acid bacteria

**DOI:** 10.1186/s12934-026-03030-w

**Published:** 2026-05-16

**Authors:** Joanna Ivy Irorita Fugaban, Emilie Munk, Saria Otani, Pascal Fourcassié, Claus Heiner Bang-Berthelsen, Egon Bech Hansen

**Affiliations:** 1 National Food Institute, Technical University of Denmark, Kemitorvet , 2800 Kgs. Lyngby, Denmark; 2 Nutritional Health and Biosciences, International Flavors & Fragrances, Brabrand, 8200 Denmark; 3 Cultures and Dairy Enzymes, Nutritional Health and Biosciences, International Flavors & Fragrances, Dange Saint Romain, 86220 France

**Keywords:** *Streptococcus thermophilus*, Cell envelope proteases, Proteolysis, *prtM*, Transformation, Strain engineering, Prolyl-peptidyl *cis*/*trans* isomerase, *prtS*, *prtP*

## Abstract

**Background:**

Cell envelope proteinases have long played a pivotal role in dairy science. However, as demand for alternative food sources grows, their application in plant-based food matrices is scarcely investigated. A deeper physiological and technological understanding of these enzymes requires efficient and effective tools tailored to this emerging sector. To date, plasmid-based recombination in *Lactococcus* spp. remains the most widely used and effective method.

**Results:**

In this study, we engineered *S. thermophilus* LMD-9 as a host for heterologous expression of protease from *Lc. cremoris*. *S. thermophilus* LMD-9 offers several advantages, including its GRAS status, efficient chromosomal gene integration via natural competence, and native machinery for functional protease production, making it a highly versatile host. Previous attempts to employ this strain yielded inactive protease due to unresolved bottlenecks; here, we characterize and overcome those challenges to establish LMD-9 as a robust system for protease expression. Exploration of potential bottlenecks highlighted the availability of intrinsic peptidyl-prolyl *cis*/*trans* isomerase (PPIase; *prtM*/*prsA*) as a key factor influencing successful enzyme expression. Three recombinant strains with genotypes: LMD-9 Δ*prtS*::*ermR*, LMD-9 Δ*prtS*::*ermR*—Ω*prtP*, and LMD-9 Δ*prtS*::*ermR*—Ω (*prtM-prtP*), were generated to test the role of *prtP*-associated PPIase. Results demonstrated that inclusion of PrtP-specific PPIase from *L. cremoris* markedly enhances protease activity. In its absence, although partially compensated by the pleiotropic PPIase in *S. thermophilus*, we observed slower growth and reduced proteolytic activity.

**Conclusions:**

These findings establish *S. thermophilus* LMD-9 as a robust chassis and alternative host for heterologous expression of cell envelope proteinases. To our knowledge, this is the first work to heterologously express active CEP enzyme in this host, and it highlights the role of PPIase availability as a key determinant of successful enzyme expression. This then provides a suitable and robust host for studying the application of CEPs in both dairy and emerging plant-based food applications.

**Supplementary Information:**

The online version contains supplementary material available at 10.1186/s12934-026-03030-w.

## Background

Cell envelope proteases (CEPs), or membrane-bound proteases, have been the forefront of various studies on starter cultures in food fermentation [1, 2]. Physiologically, it serves as the primary arsenal for the breakdown of large proteins for its host’s metabolism. However, their function extends beyond providing accessible oligopeptides to the starter strains to include their contribution to flavor development, texture, and bioavailability of nutrients in the food matrices [3, 4]. The rapid increase in the shift to alternative food sources and the rise in demand for plant-based foods have pushed the boundaries of our understanding of their functionalities into uncharted territory. As we expand innovations and food production technologies, the need for appropriate tools to uncover these frontiers becomes essential.

CEPs are among the most studied enzymes in dairy science, especially in classical dairy starter strains such as *Lactococcus* sp., *Streptococcus thermophilus*, and *Lactobacillus delbrueckii* subsp. *bulgaricus*, and to some extent, non-traditional starter strains of lactic acid bacteria (LAB) [5, 6]. Subtilisin, initially detected from *Bacillus licheniformis*, is one of the simplest well-characterized S8 proteases. Comprised of 379 amino acids (aa), which make up different domains including signal peptides, pro-peptide, and its proteolytic domain (PR) [7]. Contrary to this, serine proteases found in LAB are architecturally more complex, typically longer, with sizes between 700 and 1800 aa as surveyed by Christensen et al. [8]. Consisted of different functional domains, including its signal peptide, a longer pro-peptide, and a PR domain, which typically sandwiches a protease-associated domain (PA, also known as insert domain) identified to play a significant role in substrate selectivity. Differing from subtilisin, the PR domain followed by multiple fibronectin type-III-like (Fn) domains extending from A-domain to B-domain, wherein the former aids in substrate binding and the latter’s function is yet to be completely elucidated. Uniquely different from subtilisin is the presence of a helical domain (H-domain), which typically precedes the W-domain (cell-wall spacer), and is mostly found in lactococcal and streptococcal proteases, yet not in some other S8-like proteinases identified in other LAB [7–9].

Although our understanding of these enzymes has advanced, significant knowledge gaps remain. *In vivo* characterization of these enzymes is particularly challenging, requiring intricate and tedious techniques when expressing them in an isogenic background [10]. Key bottlenecks hinder these techniques, including host suitability, elaborate engineering techniques, low transformability, poor gene stability, and the difficulty of inserting large recombinant sequences into the host genome [11]. While attempts to heterologously express proteinase genes (*prt*P) from different LAB have been performed, myriad issues have been encountered. Some of which include the works of de Vos et al. [12] on *prtP* from *Latococcus cremoris* (prev. *Lactococcus lactis* subsp. *cremoris*) Sk11 in *Escherichia coli*, having difficulty maintaining their expression in high copy number plasmids in this host. Kok et al. [13] initially attempted to heterologously clone protease *Lc. cremoris* Wg2 (prev. *Streptococcus cremoris*) in *E. coli*, showing poor transformability and toxicity to this host, and in *B. subtilis*, although confirmed to be expressed, structural modification and incomplete expression were observed. However, homologous expression of lactococcal CEPs using plasmid vectors was found to be fully functional, stable, and conserved relative to its native form [12, 13].

Although *Lc. lactis* serves well as an isogenic host even for large gene clusters such as CEPs and exopolysaccharides [14], instability of plasmid expression remains a significant risk, attributed to various physiological and environmental factors [13, 15, 16], leading to the exploration of chromosomal integration for CEPs recombination. This, however, presented the challenge of the gene-dosage effect, yielding lower expression of proteolytic activity compared to multi-copy plasmid expression in this host [17]. The survey of prior works on heterologous gene expression of CEPs is limited. As an alternative to *Lactococcus* as a vector system, *S. thermophilus* has been the next viable host [1, 18, 19]. Its versatility as an isogenic host can be attributed to its ability to acquire exogenous genetic material when in a state of competence for transformation [19]. Regulated by its novel quorum sensing, which can easily be induced by a competence signaling peptide. Natural transformation has its array of advantages compared to plasmid-based approaches, including rapid and efficient marker-free chromosomal integration of gene inserts, as well as the capacity to incorporate large recombinant gene fragments [18, 20, 21]. It is also considered to be less tedious compared to electrotransformation-based mutagenesis assays [22, 23].

Lecomte et al. [18] used *S. thermophilus* LMD-9 as a host for the heterologous expression of *prtH* from *Lb. helveticus* CNRZ32 CIRM-BIA 103 strain. However, various issues were cataloged in their constructions, including failure to detect surface localization of *prtH* via its own anchoring motif, remedied by commissioning the intrinsic anchoring mechanism of *prtS* by retaining wild-type cell wall spacer (W) and anchoring (AN) domains. Although expression has been detected, the protease’s activity was not restored, suggesting a possible failure in enzyme maturation. Hence, in this study, we aimed to explore the role of the associated peptidyl-prolyl *cis*/*trans* isomerase (PrtM) during heterologous expression of these large enzymes in an isogenic background. Furthermore, we aimed to optimally engineer the insertion of different protease alleles into the *S. thermophilus* background for the heterologous expression of CEPs from other LAB.

## Results

### Construction of LMD-9 mutants for evaluating the role of*prtM* in heterologous gene expression of *prtP*

*S. thermophilus* LMD-9 is a well-studied strain, particularly in its application in dairy science [18, 24]. In this study, the engineering locus was identified as the *prtS* genomic island (reference sequence NC_008532.1, STER_RS06195), schematic diagram of the recombination strategy is provided in supplementary Fig. 1 (SF1). Recombination arms were designed to have at least 650 bp upstream and 680 bp downstream of the *prtS* sequences (SF1), and the recombinant fragment insertion site was defined between these two fragments. Plasmid pIL22 from *Lc. cremoris* MS22425 containing target fragments *ermR*, *prtM*, and *prtP* from *Lc. cremoris* WG2. Along with the recombination arm, target fragments were amplified, cleaned, and verified by agarose gel visualization prior to Gibson assembly. The transrtformation rate has been observed to be around 20–30%.

**Fig. 1:**
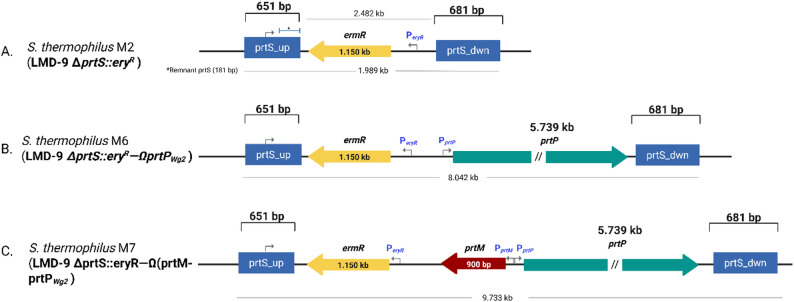
The schematic representation of the gene organization of derivative mutants from *S. thermophilus* LMD-9 was confirmed via whole genome sequence analysis. **A** *S. thermophilus* M2 as a protease KO strain (∆*prtS*::*ermR*) with a length of 1.989 kb within the recombination site from the upstream recombination arm to the downstream recombination arm, **B ***S. thermophilus* M6 with a heterologous protease gene (*prtP*) from *Lc. cremoris* Wg2 (prev. *Lc. lactis* subsp. *cremoris*), and **C** *S. thermophilus* M7, a derivative of LMD-9, with prolyl-peptidyl isomerase enzyme (maturase/*prtM*) from prtP from Wg2 with their native promoters.

Whole-genome sequence analysis revealed the gene organization of the recombinant inserts in the transformants at the target locus (Fig. 1). On the upstream recombinant arm of *prtS*, 181 bp were observed to be from the WT, of which the ribosome binding site (RBS) and promoter region are still intact. The putative signal peptide region of *prtS *was also analyzed closely, spanning 60 amino acids (aa) before termination, including an intact signal peptide from the WT. The signal peptide of *prtS* comprises 35 aa, cleaving between positions 35 and 36, as assessed by SignalP6.0. Although the next 24 aa in the pro-peptide terminate prematurely.

Upstream and downstream recombination arms (Figure SF1) facilitate gene insertion through double-crossover recombination. Identification of these recombination regions was based on Boulay et al. [25]. In *S. thermophilus* M2, removal of the fragment from the pro-peptide domain of the *prtS* and insertion of the *ermR* gene, oriented antisense relative to *prtS*. The total length of the recombinant region is approximately 1.989 kb.

For heterologous recombination of PrtP from Wg2, the coding sequence of the CEP gene, including all domains from the signal peptide (SS) to the anchoring (AN) domain of the protease, extends approximately 150 bp upstream and 80 bp downstream of the target gene to include the RBS and terminator regions. Relative to the promoter region of the *prtS* gene, *prtP* is transcribed on the positive strand as seen in Fig. 1 for strain *S. thermophilus* M6. The promoters’ regions for both *prtM* and *prtP* are included in the *prtP* insert for this version, excluding *prtM* from the beginning of its coding region. Construction for the mutant with genotype ∆*prtS*:: *ermR*-Ω (*prtM*-*prtP*_WG2_), named *S. thermophilus* M7, target fragment *prtM*-*prtP* amplified from plasmid pIL22 end to end of the terminator region of both genes (combined teal and maroon regions in Figure SF1), making sure that the intrinsic convergent promoters are in the insert fragments. The resulting recombinant fragments for M6 and M7 are 8.043 kb and 9.733 kb, respectively.

During the construction of LMD-9 derivatives, we observed series of failed inserts (Figure SF2). This observation is due to the proximity of the outermost primer downstream to a mobile genetic element identified to be from the IS3 family. Zooming in, we identified that the reverse primer for the downstream *prtS* fragment overlaps close to the IS element. This factor is worth noting as it may play a significant role in achieving the correct insertion, especially since it lies within the recombination arms, which are crucial to the double crossover.

### Phenotypes and growth of LMD-9 transformants

The expression of CEPs was analyzed by investigating the following phenotypes: acidification in milk, growth in chemically defined medium containing casein, analysis of peptides in fermented milk, and change in gene expression following a media shift from medium with casein hydrolysate to high molecular weight casein.

**Fig. 2:**
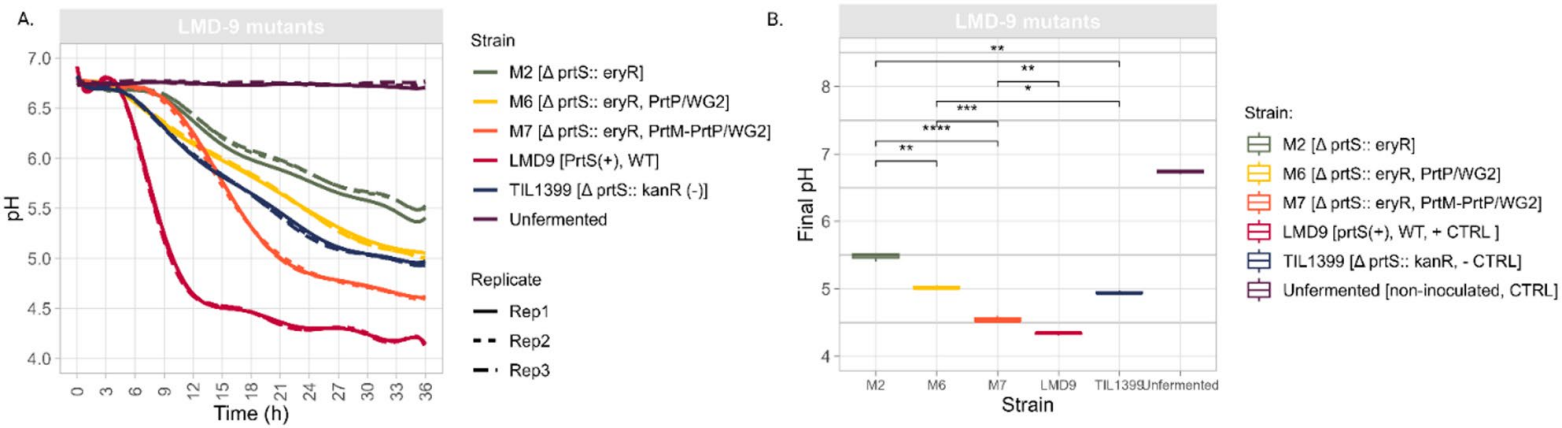
Acidification dynamics of LMD-9 derivative strains in **A** skimmed milk with 1% sucrose for 36 h at 37 °C. **B** Comparison of means of final pH after 36 h across strains. Significance levels are indicated by *, and *p*-values are marked as follows: **** if *p* ≤ 0.0001, *** if *p* ≤ 0.001, ** if *p* ≤ 0.01, * if *p* ≤ 0.05. Non-significant comparisons were left unmarked.

**Fig. 3:**
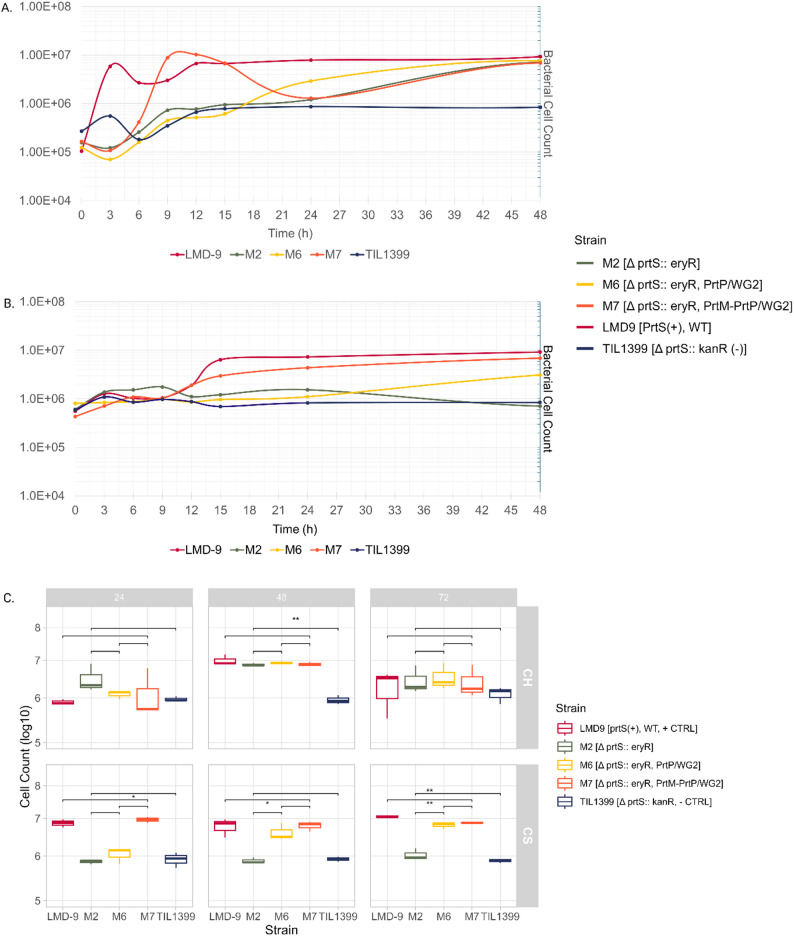
Bacterial cell counts in chemically defined medium supplemented with 1% sucrose and 1% protein source of either **A** casein hydrolysate (LMW milk proteins) and **B** casein sodium salt (HMW milk proteins), monitored every 3 h for the first 24 h and every 24 h thereafter until the 72 h. **C** Comparison of means of the bacterial cell counts measured at 24 h, 48 h, and 72 h. The unfermented control and buffer control had no cell counts recorded for all time points (data not shown). Asterisks (*) indicate significance level from pairwise t-test, applied after assessing normality and homogeneity of the dataset; pair-wise comparisons with no significant differences were left unmarked. Significance levels are marked as follows: **** if *p* ≤ 0.0001, *** if *p* ≤ 0.001, ** if *p* ≤ 0.01, and * if *p* ≤ 0.05

Acidification profiles of LMD-9 strains were assessed in milk for 36 h at 37 °C in microvolumes with a standard final inoculum of OD_600_ = 0.01. Acidification profiles are provided in Fig. 2A. The baseline pH for unfermented milk, as identified in the assay, is 6.5. WT strain LMD-9 reached pH ≈ 4.50 around 13 h (Fig. 2A). In 2B, LMD-9 reached a final acidification of around 4.3 pH. The derivative LMD-9 *prtS* knockout strain M2 had a higher pH compared to negative control *S. thermophilus* TIL1399, while M6, which lacks the associated maturase enzyme, showed a profile closer to that of the negative control. Although it did not acidify as deeply as the WT strain, mutant strain M7 with the *prtM*-*prtP*_*Wg2*_ insert was observed to be the second-best acidifier. However, it required the strain almost 23 h longer than LMD-9 to reach pH = 4.5. In summary, the acidification profiles showed that the strain M2 displayed a non-proteolytic phenotype compared to the parental strain LMD9, verifying that *prtS* has been inactivated by the insertion of *ermR*. Strains M7 and M6 acidified faster and reached lower pH values than M2, indicating expression of PrtP in *S. thermophilus* , although M6 acidified less efficiently than M7. TIL1399 reached a lower final pH than the M2 strain, despite both strains being *prtS* knockouts. Growth curve analysis in chemically defined medium supplemented with 1% protein source of either free amino acid supplemented CDM (casein hydrolysate, CH-CDM) or high molecular weight (HMW) protein (CS-CDM, casein sodium salt) was monitored every 3 h for the first 24 h, after which, samples were collected every 24 h until 72 h. Derivative mutant strains of LMD-9 grew exponentially for the first 24 h; thereafter, cell counts declined in high-free amino acid/oligopeptide environments. In HMW-supplemented CDM, negative control TIL1399 and the protease knockout strain M2 showed no significant change in bacterial cell population at 24 h, 48 h, or 72 h (Fig. 3B and C). Among protease-positive strains, the WT strain LMD-9 exhibited exponential growth between 6 h and 15 h, whereas the mutant strain M7, carrying both maturase and protease enzymes from Wg2, showed a longer lag phase than the WT strain. Although no significant differences in bacterial cell count were observed in M6, significant changes occurred within the first 24 h, corroborating the findings in CS-CDM.

The growth of strains in anamino acid -rich medium was not significantly different between mutant strains after t24 and t72 (SF3). However, at t48, there was a significant change in bacterial population between the mutant strains and LMD9 against the negative control TIL1399. Interestingly, mutant strains have the same growth patterns and tendencies in this medium. Bacterial cell count in CDM with HMW protein source showed that LMD-9 with the highest population change between time 9–12 h of ≈ 1.5 × 10^6^ cells/.h. Among the mutant strains, M7 heterologously expressing a protease with its intrinsic maturase enzyme from *Lc. cremoris* Wg2, showed the fastest rate of population change between times 12–15 h with rate of 4.0 × 10^5^ cells/h change in the MHW medium. Although *S. thermophilus* M6, with only the *prtP* gene, has the highest growth increase beyond the 24 h period after inoculation. Extended growth to 72 h (Fig. 3C) indicated that the strains are still growing up until this time reaching ≈ 2.0 × 10^6^ compared to the ≈ 4.0 × 10^6^ population of strain M7 at time 24, highest population recorded for this strain before death phase.

In casein hydrolysate-supplemented CDM, strains are expected to grow irrespective of the presence or absence of CEP. Strains M2, M6, and M7 reached the same cell count as LMD9 after 48 h. The three strains show a delay compared to LMD9, with M6 showing the largest delay. Surprisingly, TIL1399 fails to reach this level within 48 h. In the HMW casein-supplemented CDM, only strains with a CEP are supposed to grow to a high level. In this medium, strains LMD9, M7, and M6 reached high cell density at 48 h, indicating that M6 and M7 express PrtP in *S. thermophilus*, with M6 attaining this high cell density more slowly.

### Proteomics analysis

Nitrogen metabolism is crucial for the growth of microorganisms. The activities of these proteases are optimal for their energetics, and their specificity dictates the environment in which these organisms can grow [3, 26, 27]. To further investigate the necessity of the intrinsic maturase enzyme associated with these proteases, the peptidome profiles generated for each mutant were assessed. The activities of these enzymes in skimmed milk fermentation for 24 h are shown in Fig. 3.

**Fig. 4:**
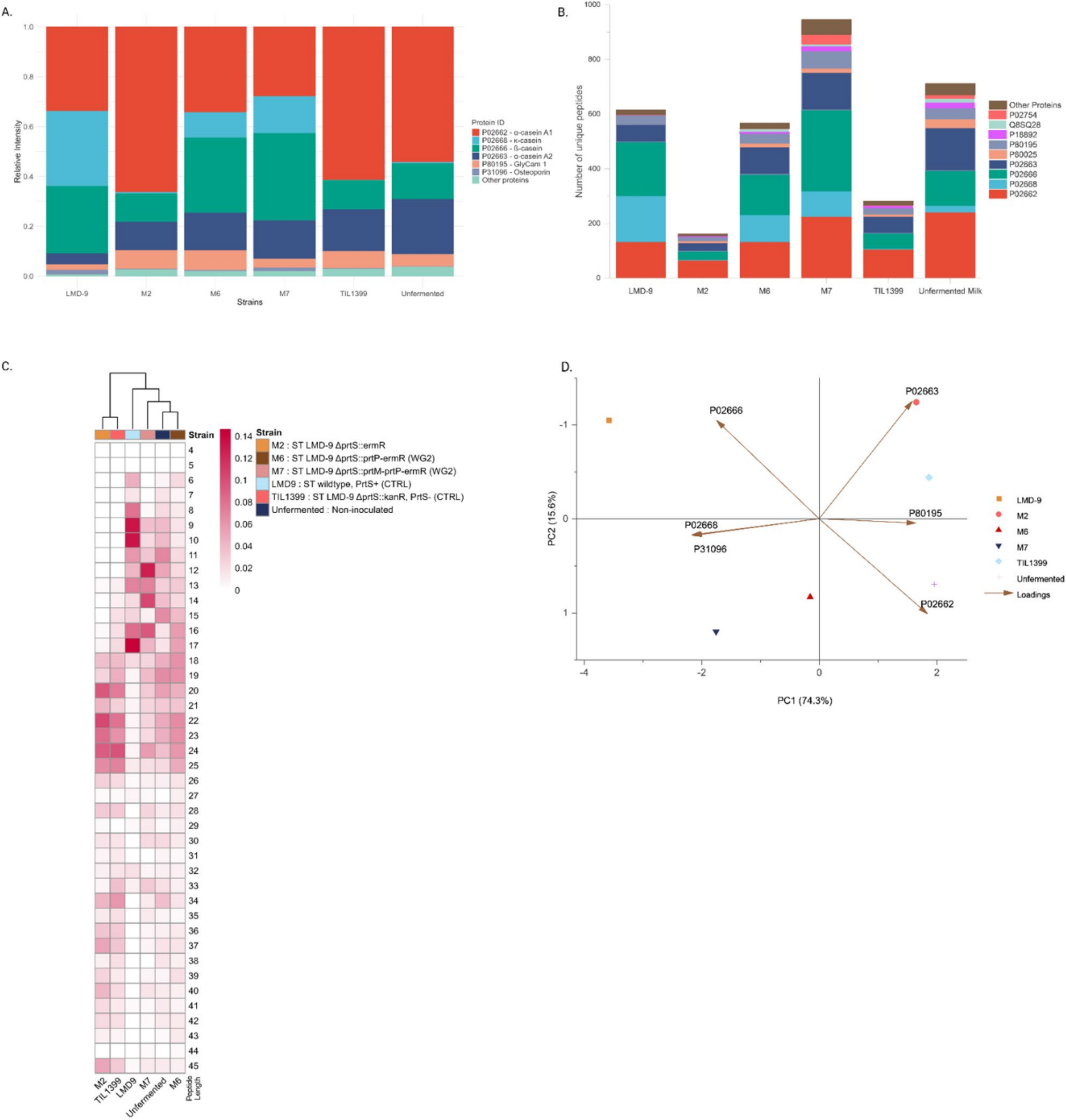
Hydrolysis profiles of LMD-9 derivative strains, showing **A** sources of identified residual peptides in the matrix, **B** number of unique peptides from the proteins, **C** peptide length distribution, and **D** PCA biplot of proteome profiles showing the effect of proteins on strain clustering, where PC1 and PC2 represent milk hydrolysis profile variances across strains, accounting for 74.3% and 15.6%, respectively. Arrows and their lengths indicate the influence of proteins and their relative contributions to clustering.

Peptide sources were identified by proteomics analysis (Fig. 4A), showing that, in LMD-9, approximately equal relative amounts of peptides originated from α_s1_-, β-, and κ-caseins, a hallmark of PrtS activity in milk proteins, showing an intermediate PI/PIII type protease [28]. The PI-type profile, typically observed in most lactococci, was exhibited by strains M7 and M6 to a lesser extent. The negative control TIL1399 and ∆*prtS* strain M2 shared a very similar profile with each other, characterized by dominance of α-_s2_ casein-derived peptides in the matrix, indicating a relative decrease in the other peptides. The number of unique peptides identified was consistentwith previous observations by Fugaban et al. [30], who compared the activities of *prtP* in *Lactococcus* sp. and *S. thermophilus prtS*; the unique peptides identified are much more diverse in lactocepins than in the latter. Focusing on mutant strains M6 and M7, the distribution of peptide sources was relatively similar; however, the way these proteins were cleaved into oligopeptides differed. Strain M6 had a more uniform set of oligopeptides identified, whereas M7 behaved more similarly to *prtP* as expressed in *Lc. cremoris* host, cleaving peptides at non-specific sites while being consistent with the optimal length that it produces as seen in Fig. 3C. The most abundant residual peptides in the matrix had of lengths of 12–16 aa, except for 15 aa length peptides, which seeminglyrepresent a preferred length for the *S. thermophilus* uptake system. Additionally, ∆*prtS* strain M2 shows a more protease negative profile compared to TIL1399, with similar peptide sources, peptide length profiles, and the number of unique peptides identified; however, this observation indicates that *S. thermophilus* LMD-9 uptake system can efficiently use peptides up to 17 aa length without requiring a protease.

Strain clustering identified that hydrolysis of both κ-caseins and β-caseins plays a significant role in the profile variances of protease-positive strains. While M6 and M7 clustered together on the same quadrant, are overall separated by the variances of their activities in κ-caseins and osteoporin. Both protease-negative strains, TIL1399 and M2, were clustered in the same quadrant, although separated by their hydrolysis profiles of α_s2_-casein.

Combining the abundance, number of peptides, and the peptide length, it can be concluded that M2 and TIL1399 are protease-deficient, as these two strains reduce the total number of peptides by consuming all peptides shorter than 18 aa. The PrtS protease of LMD-9 causes a reduction in the length of peptides rather than an increase in the number. M7 gives an increase in the number of peptides as well as a reduction in peptide length, clearly showing this strain to be proteolytic, allowing the conclusion that PrtP is expressed. For M6, the number of peptides is larger than for strain M2, indicating that PrtP is also expressed in this strain. The peptide source confirms that M6 and M7 both have a CEP of the *Lactococcus* PrtP-PI type rather than the PrtS type.

Free amino acid profiles showed that the key distinction between mutant strain M6 and M7 was the higher accumulated relative amount of Ile in M7 compared with M6. This amino acid is known as the key aa regulator for *codY.*

### Gene expression

**Fig. 5:**
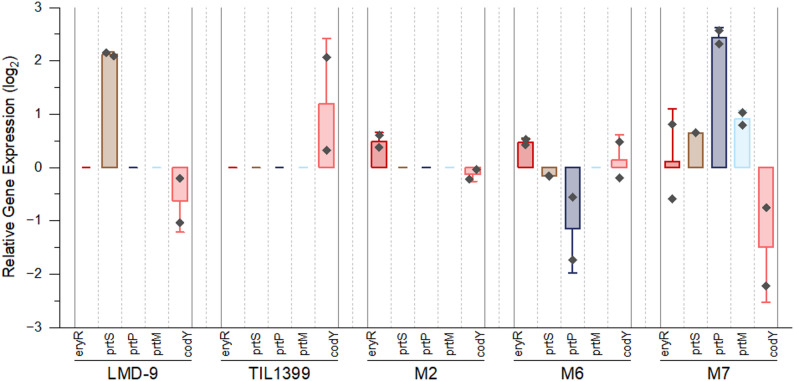
Change in relative gene expression after the medium shift experiment. The expression levels of *prt*S, *prt*P, *prtM*, *ermR*, and *cod*Y were quantified using qRT-PCR after two generations in HMW protein-supplemented CDM. Genes were normalized relative to *ldh-l* (lactate dehydrogenase) genes, which was constant across all conditions. Normalized gene expression levels were compared before (LMW-CDM) and after the medium shift.

To further assess whether the proteinase genes are expressed in the LMD-9 isogenic strains, we assessed the induction of genes during medium shift, from high FAA to low FAA environment, after two generations (Fig. 5). Expression of LMD-9 *prtS* in HMW protein CDM demonstrated repression of *codY* and induction of *prtS.* Similarly, strain M7 showed a comparable gene expression patterns, except that both *prtM* and *prtP* are induced. However, M6 showed induction of *codY* similar to TIL1399, but a repression of the *prtP* gene, contrary to M7.

A closer look at gene expression before medium shift showed that, for *prtP* expression, baseline expression in casein hydrolysate CDM had an M6:M7 ratio of  ~ 0.92:1 After the medium shift to HMW protein CDM, M7 showed a relative increase of *prtP* expression between 2- and 4-fold compared to M6.

## Discussion

*S. thermophilus* LMD-9 is a well-characterized strain for its proteolytic activity in dairy [29] and milk alternatives [25]. Available techniques for the genetic engineering of this strain have been well explored. Itscapacity for natural transformation through competence induction, as well as its uptake of large genetic material , makes it a good host candidate. This then allows *S. thermophilus* to be a valuable tool for providing an isogenic background, especially for inserting large recombinant inserts, as well as for the possibility of marker-free integration in a food-grade host [19].

A less tedious approach to isolating CEP activities would be particularly valuable when characterizing these enzymes in plant proteins, which are generally complex and for which assay systems remain less established than for milk proteins. In a previous study, we identified multiple CEP homologs from various LAB strains [30] that showed distinct activity in plant proteins. Thus, having a host suited for a plug-and-play approach to characterize these proteases would be of clear interest for future studies targeting plant-based substrates. While the present work does not directly assess the hydrolysis of plant proteins, the isogenic host developed here provides a practical framework for evaluating promising CEP candidates. Having identified that *S. thermophilus* LMD-9 is a robust strain that can grow in plant-based milk alternatives [25, 30, 31].

The construction of LMD-9∆*prtS*:: *ermR* (M2) knock out strain followed Boulay et al. [25]. Focusing on the *prtS* genomic island as a recombinant locus, wherein modifications from their work include shortening the remnant *prtS* sequence, serving as recombinant arms, to abolish residual proteolysis activity from the WT strain. To facilitate efficient heterologous recombination, retention of a downstream arm ≥ 600 bp, which positions the reverse primer binding adjacent to an IS element (see Figure SF1). Some of our constructions (Figure SF2) exhibited multiple insertions close to an IS element of a similar family located downstream of *prtS*, likely due to this proximity.

The design for the recombination work in this study was based on the various limitations identified in the first reported attempt at heterologous gene expression of a LAB protease in *S. thermophilus* LMD-9 by Lecomte et al. [18]. Their work on *prtH* from *Lb. helveticus* in this background encountered various issues, ultimately leading to inactive protease expression in the mutant strains, despite gene expression being detected. One of the significant points they made is the importance of a protease anchoring mechanism similar to that of the host. Lecomte & co. remedied this issue by swapping the intrinsic ANdomain of PrtH with PrtS, leading to higher gene expression of *prtH* in the mutant, although proteolysis was still not observed. This led us to use PrtP from *Lc. cremoris* Wg2 as an insert to optimize recombination of protease alleles in *S. thermophilus* LMD-9, apart from it being well studied, allowing us to delimit possible issues and pinpoint bottlenecks while designing a suitable isogenic strain. Tackling the last remaining question posed by their work, of the necessity of intrinsic maturase enzyme associated with *prtP* when doing a heterologous gene expression in *S. thermophilus*. To proceed, we performed an initial follow-through bioinformatics analysis on the inserted *prtH* of *Lb. helveticus* CNRZ32 used in their study (Supplementary Material, SF2), identifying that the protease they used has a maturase enzyme divergently oriented from it, a common observation for *prtM*-*prtP* gene clusters, especially those characterized from *Lc. lactis* strains [32, 33]. Furthermore, we also searched for similar peptidyl-prolyl *cis*/*trans* isomerase (PPIase) native to *S. thermophilus* LMD-9 (WGS reference sequence NC_008532.1, locus tag STER_RS02415). The construction of LMD-9 ∆*prtS*::*ermR*-Ω*prtP*_Wg2_ (M6) strategy for this mutant includes insertion of the *prtP* gene starting from the upstream region of the coding sequence, which comprises the native promoters of *prtM* and *prtP*, resulting in an intergenic region of 534 bp length PrtP promoter in M6 mutant strains was annotated based on the sequence analysis using ProPR v2.0 and confirmed by BPROM webservers (see Fig. 1, predicted promoter region SF3). Orientation of *prtP* and *ermR* genes was adapted from the donor plasmid pIL22 from *Lc. lactis* MS22425 (SF1). Similarly, the construction of recombination inserts for mutant strain M7 with genotype LMD-9 ∆*prtS*::*ermR*-Ω (*prtM*_*Wg2*_-*prtP*_Wg2_), included the intrinsic promoters of both genes as well as intrinsic terminator regions.

Phenotypic characterization of the activity of the lactococcal protease expressed in *S. thermophilus* was performed in skimmed milk with 1% sucrose (w/v). Acidification profiles showed that the WT strain had the fastest acidification rate, followed by mutant strain M7 (data not shown, see Fig. 2). M6 shared a profile closer to TIL1399 after 36 h of fermentation. Growth profiling of the strains in chemically defined medium with HMW protein showed that both the WT strain and M7 can grow up to 10^7^ cells/mL, while M6 demonstrates a longer lag phase, reaching a final population of ~4.0 × 10^6^ cells. In milk, it has been demonstrated that microbial cells can grow up to ~10^8^ without requiring their own protease facilitated by available oligopeptides. However, increasing cell density beyond this point requires functional proteolytic system [34–36]. This might corroborate the observed acidification profiles of M6 and TIL1399 in milk and HMW protein supplemented CDM. As expected, knockout strain M2 did not acidify milk lower than pH 5.5 and shared a similar growth profile with TIL1399 in HMW protein supplemented CDM. To further assess the activities of PrtP with and without its intrinsic PPIase, the hydrolysis activities of the mutants in milk proteins were profiled by proteomics analysis. A survey for oligopeptide sources showed that the unfermented milk has an abundance of α_s1_-, β-, and α_s2_-caseins, respectively, in their compositional ratio, between sizes 4–45 aa. Negative strains showed a similar profile as unfermented milk with regard to this parameter. WT protease, PrtS, with an overall higher affinity for κ-caseins relative to PrtP , showed close to an equal relative amount of peptides from β-, α_s1_-, and κ-caseins. Mutant strains M6 and M7, both having lactocepin, share almost identical peptide sources, except that M7 has more peptides cataloged from κ- and β-caseins than M6, which might indicate that the protease of M7 is much more active than that of M6. This is further supported by the higher number of unique peptides and the peptide length distribution of M7 fermentate, skewing the distribution to its preferred length of 9–16 aa [3]. PCA plot analysis identified that hydrolysis profile variances between M6 and M7 are strongly affected by β-caseins (P02666) and κ-caseins (P02668).

Lecomte et al. [18] noted that, despite detecting expression, their constructs produced and accumulated zymogenic PrtH . We therefore hypothesized that complementing the missing maturase enzyme with the pleiotropic PPIase from LMD-9 would restore the protease activity of M6. Although the complete maturation process of PrtS and its association with the universal PPIase found in *S. thermophilus* remains unresolved [37], a parallel study by Ikolo et al. [38] showed that in *S. equi*, deletion of *prsA*/*prtM* diminishes virulence and disrupts N-metabolism-associated proteins, including identified putative S8-like proteases (SEQ1800, annotated by Holden et al. [39].), sortase A (SEQ1171 [39], and Opp proteins [38].

In this work, the presence of a co‑localized maturase lipoprotein, may suggest the possibility of compensation, provides a more favorable physiological state for a functional enzyme by facilitating efficient expression, folding, translocation, and activation. However, several factors that may affect the expression of the gene insert in M6 should be further considered. Additionally, it is imperative to highlight that the *prtM*-*prtP* promoter regions in M6 may also cause transcriptional interference, especially if the *prtM* gene is removed. Transcriptional activity of divergent promoters, explored by Wang et al. [40], showed that an aggressive/strong promoter of the TyrR protein might occupy RNA polymerases, limiting binding to the weaker/sensitive promoter of the *aroP* gene in *E. coli*. Also, this highlights the unique promoter orientations of *prtM* and *prtP* in *Lactococcus* sp., an overlapping divergent promoter (i.e. *Lc. cremoris* Sk11). Overlap is found to play a role in the co-regulation of these two genes [33]. The absence of *prtM* in M6 may be a strong factor affecting the protease’s activity in this version. Hence, future constructs with well-organized inserts with an isogenic promoter would help to eliminate this factor. Although we attempted to rule out transcriptional occlusion as the cause of interference by extending the incubation period in milk fermentation to 36 h to account for M6’s prolonged lag phase in HMW-CDM, M6 still exhibited a profile similar to TIL1399 rather than M7. This might explain the unexpected gene expression levels observed in M6 during medium shift experiments. Collectively, these observations suggest that the absence of the cognate PrtM remains the primary bottleneck to the protease maturation in M6. While pleiotropic PPIase of LMD-9 may partially substitute for PrtM, its capacity is evidently limited, as reflected by the longer lag phase and suboptimal milk protein hydrolysis observed in M6, which performed better than the protease-negative strain M2 and TIL1399, yet evidently inferior to M7. It is also worth noting that the native promoters of *prtM* and *prtP* were deliberately retained in these constructs, as they partially overlap and are subject to identical transcriptional control by CodY , the global regulator of nitrogen metabolism conserved across lactic acid bacteria [33]. Altering these promoters might complicate the known regulatory mechanism and affect the experimental conditions under which their expression was measured in this study. Most LAB lack complete biosynthetic pathways for numerous amino acids, ranging from 10 to 14 aa, like in *Lb. helveticus* [41], or 7–8 aa, as in *Lc. lactis* MG1363 [33], making these organisms auxotrophic and reliant on peptide uptake and extracellular proteolysis for their nutritional needs. However, aside from their role in the growth of these organisms, branched-chain amino acids (BCAAs) and the global transcriptional regulator CodY have a tight interplay in regulating protease activity in proteolytic LAB [42, 43]. This provides a mechanistic basis for the contrasting *prtP* expression patterns observed between M6 and M7 upon media shift. Free amino acid profiles showed that M7 accumulated higher relative amounts of Ile compared to M6, which is known as the key effector of CodY [44, 45]. The higher Ile pool in M7, therefore, reflects a more active proteolytic system, where efficient hydrolysis of HMW proteins by the properly matured protease releases greater amounts of amino acids, including Ile, compared to M6.

## Conclusions

This study highlights the role of a *prtP*‑specific PPIase in facilitating the efficient expression of an active heterologous protease in *S. thermophilus* LMD‑9. The host’s pleiotropic PPIase appears to partially compensate for the missing maturase enzyme (PrtM) from *Lc. cremoris* Wg2, although it does not fully match PrtM’s native activity, as reflected by the phenotypic and metabolic divergence among mutant strains. Moreover, this finding provides insight into a possible role for the intrinsic PPIase in the maturation and activation of PrtS in *S. thermophilus*, further supporting the role of the maturase enzyme in CEP processing. Finally, by addressing known challenges in using *S. thermophilus* as a host for heterologous LAB protease expression, our work offers an alternative microbial engineering tool for characterizing large, surface‑anchored proteins such as CEPs, delivering stable gene inserts via chromosomal integration and streamlining recombination through natural transformation.

## Methods

### Bacterial strains, growth conditions, and target fragment preparation

Strains used in this work are listed in Table 1. The strains were cultured in M17 broth (Difco, USA) with 0.5% sucrose (w/v) or 0.5% glucose (w/v) (Sigma Aldrich, USA) or on corresponding agar plates with 1.5% agar (w/v). Incubation temperatures used for *S. thermophilus* and * Lc. cremoris* strains are 37 °C and 30 °C, respectively. Additionally, corresponding antibiotics were used at the following concentrations: erythromycin (5 µg/mL) and kanamycin (1000 µg/mL) (all from Sigma-Aldrich).

**Table 1:** Bacterial strains used in this work

Strains and plasmids	Description	Source/reference
Strains		
* S. thermophilus* LMD-9	WT, prtS^+^ (host)	INRAE
* S. thermophilus* TIL1399	LMD-9 ∆*prt*S::kan^R^	Rul, F. [INRAE, personal communication]
* Lc. cremoris* subsp. *cremoris* MS22418	*Lc. lactis* MG 1363 with plasmid pIL18, *ermR*,* prtP*^*−*^	[3]
* Lc. cremoris* subsp. *cremoris* MS22421	*Lc. lactis* Mg1363 with plasmid pIL18, *ermR*,* prtM*,* prtP*^*+*^ (PrtP_SK11_)	[3]
* Lc. cremoris* subsp. *cremoris* MS22425	*Lc. lactis* Mg1363 with plasmid pIL18, *ermR*,* prtM*,* prtP*^*+*^ (PrtP_WG2_)	[3]
* S. thermophilus* M2	LMD-9 ∆*prt*S::*ermR*	This study
* S. thermophilus* M6	LMD-9 ∆*prt*S::*ermR*-Ω*prtP*	This study
* S. thermophilus* M7	LMD-9 ∆*prt*S::*ermR*-Ω(*prt*M, *prt*P)	This study

Strains used were grown overnight in liquid cultures. Cells were harvested by centrifugation (8000 ×*g*, 4 °C, 10 min) for DNA or plasmid extraction. Genomic DNA of strains used was extracted using DNeasy^®^ UltraClean Microbial Kit (Qiagen, Germany) while plasmids were extracted using Plasmid Mini AX kit (A&A Biotechnology) following the manufacturer’s instructions. For the lysis step for plasmid extraction, 50 units/mL mutanolysin and 10 mg/µL of lysozyme (both from A&A Biotechnology) were added to the buffer, followed by incubation at 50 °C for 30 min. The obtained genetic material were quantified using Qubit Broad Range dsDNA assay (Thermo Fisher Scientific, Germany).

Target fragments were PCR-amplified (Applied Biosystems, Thermo Fisher Scientific) using the primers described in Table ST2. Cycling conditions are provided in Supplementary Table 1. PCR products were cleaned up using MiniElute PCR Purification Kit (Qiagen) for fragments ≤ 4 kb and gel-based purification in 0.8% (w/v) agarose gel with SYBR Safe DNA gel stain (Thermo Fisher Scientific) using QIAquick Gel Cleanup Kit (Qiagen) following the manufacturer’s instructions. Additional washing steps were performed to completely remove salt before the Gibson assembly. Construction of recombinant fragments was done by following recommended incubation conditions for Gibson Assembly Master Mix (New England Biolabs, Germany) for the cleaned target DNA obtained. Purified recombinant linear DNA fragments were stored at −20 °C until use.

### Transformation of *S. thermophilus* strains

Natural transformation experiment were carried out as suggested by Dorrazehi et al. [46] with some modifications. Briefly, host *S. thermophilus* LMD-9 and donor strains (Table 1) were grown overnight in a chemically defined medium as described by Lecomte et al., [19]. Bacterial cells adjusted to ≈ 10^9^ CFU/mL were inoculated into 1 mL of skimmed milk (0.1% fat, Arla) with 1 µM *comS* inducer peptide (XIP peptide, LPY-FAGCL). Cells were incubated for 75 min at 37 °C. Milk culture samples of 300 µL were made, and at most 10% (v/v) insert recombinant linear dsDNA fragments were added to obtain a final concentration of at least 1 pmol/mL to a maximum of 10 pmol/mL. Subsequently, samples were incubated for 3 h to facilitate transformation before plating on M17 agar containing 1% glucose (w/v) and the appropriate antibiotics. Plates were incubated for 24–48 h on the same conditions. Single colonies of putative mutants were re-streaked twice on gM17 with 5 µg/mL erythromycin prior to transferring them into the liquid medium of similar composition. A PCR-based screening assay was carried out to identify successful transformants. Positive strains were purified by re-streak method in sM17 agar plates with erythromycin.

### Genome sequencing of LMD-9 transformants

Total DNA was extracted using the Quick-DNA HMW Magbead Kit (Zymo Research, USA) following the manufacturer’s instructions and minor modifications: starting material of 170 ± 5 mg and resuspension in 200 µL DNA/RNA shield R1100, followed by incubation for 10 min, during microbial lysis, treatment was done with 100 uL lysozyme and lysostaphin, and during DNA purification the incubation in step 3 was performed for 15 min. DNA yield was measured using the Qubit 4 Fluorometer (Invitrogen, Thermo Fisher Scientific), and DNA was stored at 4 °C until library preparation. Approximately 1 µg DNA input from each sample was used for library preparation with the Ligation sequencing gDNA Native Barcoding Kit 24 V14 (Oxford Nanopore Technologies, UK) (protocol version 06/01/2023) following the manufacturer’s instructions with minor modifications: increased incubation times during end prep to 10 min, and barcode and adaptor ligation steps to 40 min. All libraries were loaded on a FLO-PRO114M flow cell. Sequencing was performed for 72 h, and reads were basecalled using Guppy Basecaller (v7.2.13) [47] with super accurate base calling in real time. Low-quality reads were filtered using the default setting on MinKNOW with a score of < 9 and read lengths < 200 bp. Assemblies and quality check: Genomes were assembled using Flye v2.9 with polished with medaka v1.7.2 (https://github.com/nanoporetech/medaka).

Initial annotations were performed in RAST server (http://rast.nmpdr.org) [48]. Identified target loci/putative target genes were also manually searched using BLAST and sequences were aligned by MAFFT algorithm (https://www.ebi.ac.uk/jdispatcher/msa/mafft) [49]. All other sequence analyses and manual annotations for gene boundaries were carried out in CLC Main workbench v22.0.2.

Identification of signal sequence and cleavage sites was performed in signalP6.0 (https://services.healthtech.dtu.dk/services/SignalP-6.0/) [50] for the protease sequences. Promoter regions were annotated based on the calculation obtained from BProm server (http://www.softberry.com/berry.phtml?topic=bprom&group=programs&subgroup=gfindb) [51], further confirmed by ProPr: Prokaryote Promoter Prediction v2.0 (http://propr.molgenrug.nl/). Family identification of mobile genetic elements was performed in ISFinder (https://isfinder.biotoul.fr/) [52].

### Phenotyping and growth experiments of LMD-9 transformants

#### Inoculum preparation

Selected mutants were grown o/n in liquid cultures using M17 with 1% sucrose (w/v). Cells were harvested by centrifugation (8 000 × g, 10 min, 4 °C) and washed twice using phosphate-buffered saline (1×, Oxoid^™^, Thermo Fisher Scientific). Cells were resuspended in the same buffer and adjusted to a final OD of 0.5 (600 nm) and stored in 50:50 (v/v) sterile PBS: glycerol (Sigm-Aldrich) at −80 °C in aliquot of 30 µL. Prepared inoculum were used to all subsequent experiments. Similar procedures were followed to prepare control strains used in this study.

#### Acidification in milk

Acidification dynamics of *S. thermophilus *LMD-9 mutants and controls were monitored using color-based acidification assay following Poulsen et al. [53] for 36 h at 37 °C. Medium was prepared by pasteurizing skimmed milk (1% fat, Arla, Denmark) at 99 °C, 10 min, cooled down and store at 4 °C before used. Milk was added with 1% (w/v) sucrose, 5 mM of formic acid (Sigma Aldrich), and 5% (v/v) indicator dye. Previously prepared inoculants were added to a final OD_600_ = 0.5. Experiment was carried out in triplicates.

#### Bacterial cell count in chemically defined medium (CDM)

10mL of CDM designed by Hernandez-Valdez et al. [54] with some modifications including replacement of protein sources to either 1% (w/v) casein hydrolysate or 1% (w/v) casein sodium salt (both from Sigma-Aldrich) or 1% (w/v) sucrose were prepared for each strain in triplicate. Inoculants were added to a final OD_600_ of 0.005 and incubated at 37 °C. 1 mL of samples were obtained every 3 h for the first 24 h followed by sampling at time 48 h and 72 h. Samples were immediately processed for quantification.

Bacterial cells were quantified by flowcytometry using MACSQuant^®^ VYB flow cytometry (Milteyi Biotec, Germany). The stop buffer was prepared using sterile 1× PBS (Oxoid^™^) with 1 µg/mL Hoechst 33342 dye (Sigma-Aldrich) and 250 µg/mL chloramphenicol (Sigma-Aldrich). The stop buffer was filtered (0.22 μm) prior to use. Samples were drawn from each point and were diluted and fixed using the stop buffer up up to 10^3^ dilution factor (df) in a 96-well microtiter plate (Corning Inc., USA). Prepared plates are incubated at 37 °C for 45 min prior to flow cytometry analysis. Data analysis was performed in MACSQuantify v. 2.13.3 (Miltenyi Biotec).

#### Proteomics analysis

Pasteurized skimmed milk with 1% (w/v) sucrose was fermented with the mutants and control strains inoculated to a final concentration of OD600 = 0.01. Inoculated milk samples were incubated at 37 °C for 24 h prior to analysis. Samples were kept at −80 °C until processing.

LC-MS/MS sample preparation and run were performed as described by Genet et al. [55]. Data analysis was performed in MaxQuant (v 2.4.9.0, Max Planck Institute of Biochemistry, Germany). Parameters and FASTA files of curated milk protein libraries used for running the experiments are uploaded to: https://www.computerome.dk/. Default parameters were used, except for the following modifications: unspecific digestion, peptide length minimum and maximum length of 4 to 45 aa, maximum mass 6.60 kDa. Variable modifications settings include, oxidation (methionine residues), acetyl (Protein N-term), and phospho ST, with fixed modification of carbamidomethyl (cysteine residues). The false discovery rate (FDR) is set to 0.01 at peptide and protein level.

#### Free amino acid profiles

Total free amino acid profiles of the strains were evaluated in CDM with 1% (w/v) casein sodium salt and 1% (w/v) sucrose. Fermentation was performed by using inoculum size of final OD600 = 0.01. Samples from the batch fermentation were prepared for FAA analysis as follows: for each fermented sample, 500 µL was solubilized in 100 mM ammonium formate in water (Sigma-Aldrich) 1:5 (v/v) ratio and centrifuged for 10 min at 15 000 × g at 4 °C. Clear supernatant was obtained and filtered using 0.2 μm membrane filters (Sartorius, France). Sample volumes of 2 µL were injected and were separated and analyzed using BioZen^™^ 2.6 μm Glycan (Phenomenex, USA) 100 × 2.1 mm LC column with 10 mM ammonium formate in ACN as mobile phase. Each sample was measured for 16 min with a 0.5 mL/min flow rate.

### Medium shift experiment

Bacterial cells from an overnight cultures of mutant strains *S. thermophilus* M2, M6, and M7 and control strains *S. thermophilus* LMD-9 and TIL1399 in M17 with 1% (w/v) sucrose were collected by centrifugation (8000 ×*g*, 10 min, 4 °C). Supernatants were discarded and cells were washed twice in sterile cold 1× PBS and cell densities were measured in OD_600_.

CDM with 1% sucrose (Sigma-Aldrich) and 1% protein source of either casein hydrolysate or casein sodium salt were prepared. The initial medium was pre-incubated CDM-casein hydrolysate (37 °C, 1 h). Samples of 2 mL cultures were prepared and heavily inoculated (final OD600= 1) with each strain and incubated for 2.75 h to obtain at least two generations. Cells were then collected and washed as previously described followed by resuspending the cells into pre-incubated CDM-casein sodium salt (37 °C) with the same volume and incubated in the same conditions as the initial medium. All are carried out in three independent replicates.

Bacterial cells after the second medium shift were collected and distributed into 500 mL aliquots and preserved for RNA extraction using RNA protect (Qiagen) reagent for cell pellet preservation following the manufacturer’s protocol. Cell pellets are stored at −80 °C until subsequent experiments.

### Gene expression

#### RNA extraction and cDNA synthesis

Previously collected cells from medium shift experiment were used for RNA extraction using RNeasy Mini Kit (Qiagen) following the manufacturer’s suggestion with some modifications. Briefly, cells lysis was performed by resuspending the cells in 350 µL RLT buffer with 7 µL β-mercaptoethanol (Sigma-Alrdich) prior to bead beating (60 s, high speed, cold rack, Qiagen). After the lysis step manufacturer’s suggested RNA extraction protocol was carried out. RNA extracts were eluted to a final volume of 30 µL. Concentration was quantified using Qubit HS RNA assay and quality was assessed using NanoDrop Spectrophotometer (Thermo Fisher Scientific).

Immediately, reverse transcription of collected RNA to cDNA was carried out using Invitrogen^™^ SuperScript^™^ IV VILO^™^ master mix with ezDNase^™^ treatment (Thermo Fisher Scientific) following suggested protocol. For each independent replicate, 100 ng of RNA samples were used and DNase treatment was added to remove genomic DNA. No reverse transcriptase is used as a negative control. Synthesized cDNA were quantified using QUBIT HS DNA assay and stored at −20 °C until use.

#### Quantitative PCR and data analysis

Primers used for gene expression are listed in Supplementary Material (ST1). Briefly, qPCR analysis was performed using the PowerUp ^™^ SYBR^™^ Green master mix kit (Thermo Fisher Scientific) using 5 ng of cDNA in final reaction volume of 18µL with 4µM corresponding forward and reverse primers. Quantification was performed in 384-well plateswith no-RT control as negative control. Real-time qPCR assay was performed in QuantStudio^™^ 5 Real-Time PCR System (Applied Biosystems, Thermo Fisher Scientific) using standard two-step PCR protocol.

Melting curve analysis was carried out for all assays in QuantStudio Design & Analysis Software (Applied Biosystems). Relative expression levels were quantified using comparative critical threshold method (2^−∆∆Ct^) values by using reference gene *ldh*L as a reference to quantify relative expression of *prtS*, *ermR*, *prtP*, *prtM*, and *codY*. Primer information as listed in (Table ST1).

### Data handling and analysis

Data were analyzed and visualized in R (R Core Team, 2021) or OriginPro 2024. Statistical analysis of acidification and growth curves included testing for normality using the Shapiro-Wilk Test and homogeneity using Levene’s test, with time and medium as factors for corresponding experiments. Comparison of means was performed by Student’s t-test for pair-wise comparison.

## Supplementary Information

Supplementary Material 1.

## Data Availability

All data generated or analyzed in this work are included in this published article and its supplementary information. *S. thermophilus* LMD-9 was obtained as a commercial culture. The strains used in this work and the generated mutants can be provided under a material transfer agreement (MTA) and will be considered on a case-by-case basis. Raw datasets are available from the corresponding author upon reasonable request.

## Abbreviations

CEP(s): Cell envelope proteinase (s)
PPIase(s): Peptidyl prolyl cis/trans isomerase (s)
