# Supplementary material for: Engineering Streptococcus thermophilus for heterologous gene expression of cell envelope proteases from lactic acid bacteria
Source: Microb Cell Fact. 2026 May 16;25:162. doi: 10.1186/s12934-026-03030-w (PMC13374181; doi:10.1186/s12934-026-03030-w)
Supplement: Supplementary file 1 — Supplementary Material 1. [file 12934_2026_3030_MOESM1_ESM.docx]

Supplementary Materials:

Table ST 1. Primers used in this work

| **Primer** | **Description (Donor strain)** | **Sequence** | **Amplicon Size** | **Reference** |
| --- | --- | --- | --- | --- |
| *Construction of LMD-9 derivatives* | | | | |
| PrtS_UpF1 | Recombinant arm upstream *prt*S (LMD-9) | TGGTAAGCACGTAGACCTTTAG | 651 bp | This study |
| PrtS_UpR1 |  | GCTTCCACCTTAGTCTCTAC |  |  |
| PrtS_DwnF1 | Recombinant arm upstream prtS (LMD-9) | TCGTTCAGCACAACTTGTATTG | 681 bp | This study |
| PrtS_DwnR1 |  | GTTGGTTATACTTCTTCCTTGTG |  |  |
| PrtS_EryF | *eryR, prtM, prtP* (MS22418) | GTAGAGACTAAGGTGGAAGCTGTAGCACCTGAAGTCAG | 1.150 kb | This study |
| PrtS_EryR |  | ATACAAGTTGTGCTGAACGATAATGGGAGATAAGACGG |  |  |
| PrtP_EryR | paired with PrtS_eryF (MS22418) | AAGCATTTCAGAGGAGACCGAATCGTAATGGGAGATAAGACGG | 5. 96 kb | This study |
| PrtP _F | prtP (MS22425) | CGATTCGGTCTCCTCTGAAATG | 5.78 kb | This study |
| PrtP_R |  | GTTCAATACAAGTTGTGCTGAACGACAAAGTCATCAAACCTCTG |  |  |
| *Gene Expression* |  |  | References | |
| ldh-l_F | *ldh-l* | TAAAGCTATCCTTGACGATGAA | Gardan et al. (2009) | |
| ldh-l_R |  | ACAATAGCAGGTTGACCGATAA |  |  |
| Ery_GE_F | *eryR* | CTGACGATAAGTTGAATAGATGAC | This study | |
| Ery_GE_R |  | CAAAAATATAAAATATTCTCAAAACTT |  |  |
| PrtM _GE_F | *prtM* | TCGATGAAGAAAAAAATGCGCC | This study | |
| PrtM _GE_R |  | TGTCGTTGGTGACTGTTTGAG |  |  |
| PrtP_GE_F | *prtP* | AAAGTGGAGGATATTGGATGCA | This study | |
| PrtP_GE_R |  | GCGAGTGATGATCCCTTAGTTT |  |  |
| PrtS_GE_F | *prtS* | GAGGAAAAGTTATGAAAAAGAAAGAAACT | This study | |
| PrtS_GE_R |  | GTGCTACAGCTTCCACCTTAG |  |  |
| codY_F |  | GTCCATTAGCAATCTTCCCAGA | Cretenet et al. (2014) | |
| codY_R | *codY* | CCACATGATAAATGTTCCCAAAC |  |  |

Primer overhangs corresponding to overlapping sequences of the adjacent gene insert are marked as **red** text. Amplicon size generated for gene expression have a size of 138 for all genes except for *ldhL* with140 bp amplicon size.


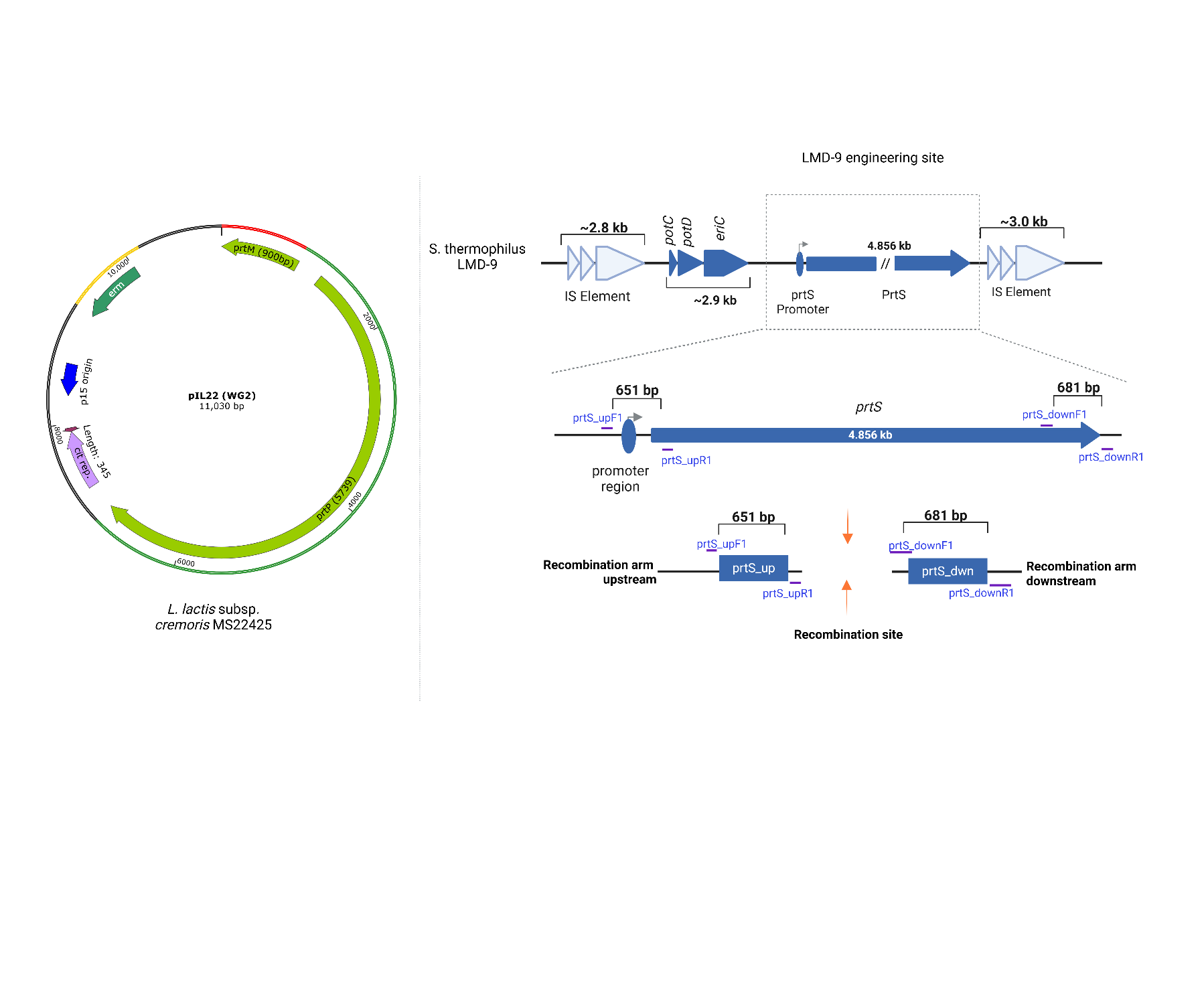


Figure SF1: Schematic diagram of LMD-9 engineering. Plasmid contains protease genes, prolyl-peptidyl cis/trans isomerase (*prt*M), *prt*P, and *ery*^R^ genes from *Lc. lactis* MS22425. Insert fragments from pIL22 for the recombination are represented by color-coded semi-circles including yellow for *ermR*, green for *prtP*_Wg2_, and red+green region for *prtM-prtP* insert fragments. Engineering locus identified as the prtS genomic island, retaining sequences for recombination arms prtS_up and prtS_dwn to facilitate double-cross over.


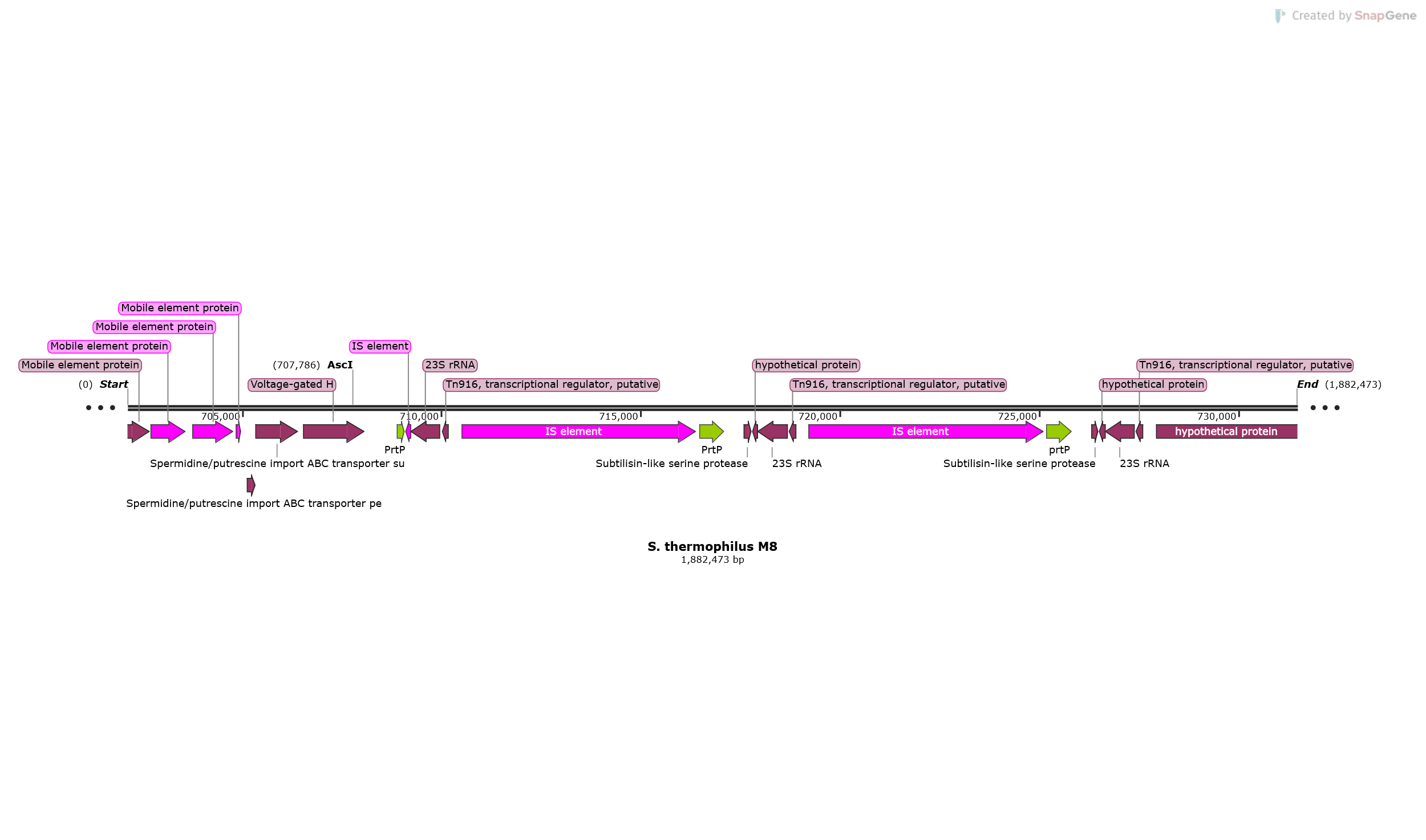


Figure SF2: Genome map of *S. thermophilus* M8 showing multiple *prtP* gene inserts outside the recombination site. Sandwiched by IS3-type insertion sequence annotated by RAST server. Recombination arm from the *prtS* is marked as “Subtilisin-like serine protease”.


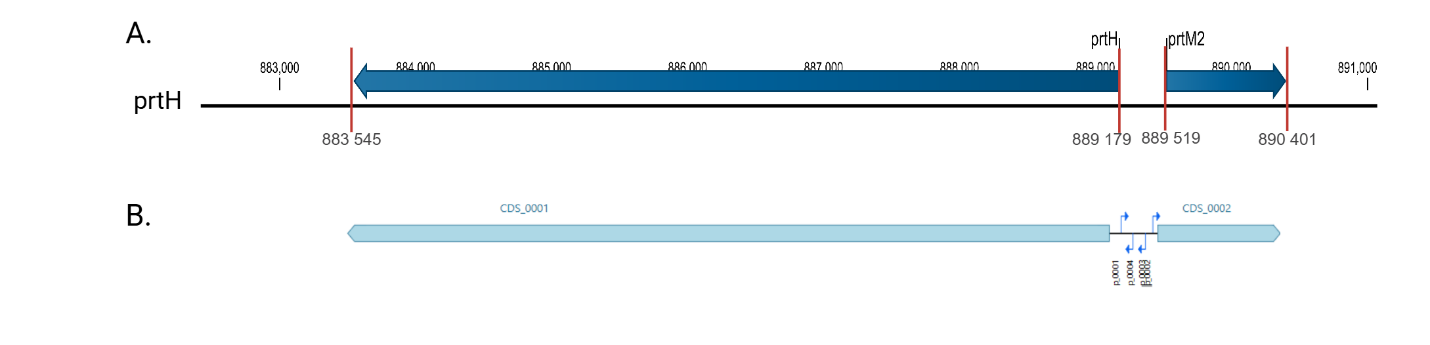


Figure SF3: Search and Mapping of proteases and PPIase found in *Lb. helveticus* CNRZ32. Whole genome sequence was accessed in NCBI genome database (NC_021744.1) searched against all four characterized identified proteases including prtH (accession no. AF133727.1, gene engineered by Lecomte et al., 2014), prtH2 (accession no, DQ826130, Smeianov et al., 2007), prtH3 (HQ602769.1), and prtH4 (HQ602770.1). Manual search for the *prtM* gene using similar gene from Sk11 (uniProt: Q02VE3). (A) Engineered prtH gene of Lecomte et al. (2014). (B) Confirmation of promoters from PePPER prediction identified probable promoter site and confirmed by BProm identifying overlapping promoters of prtH and prtM2. Scales are not representative of exact gene boundaries.
